# Effect of COVID-19 on Kidney Graft Function One Year after Onset

**DOI:** 10.3390/medicina60010026

**Published:** 2023-12-23

**Authors:** Agnieszka Malinowska, Jakub Ruszkowski, Marta Muchlado, Zuzanna Ślizień, Zbigniew Heleniak, Aleksandra Parczewska, Katarzyna Kanclerz, Bogdan Biedunkiewicz, Leszek Tylicki, Ewa Król, Alicja Dębska-Ślizień

**Affiliations:** 1 Department of Nephrology Transplantology and Internal Medicine, Medical University of Gdansk, 80-952 Gdansk, Poland; 2 7th Naval Hospital in Gdansk, 80-305 Gdansk, Poland; 3 Department of Occupational, Metabolic and Internal Diseases, Medical University of Gdansk, 81-519 Gdynia, Poland

**Keywords:** COVID-19, graft function, kidney transplant recipients

## Abstract

*Background and Objectives:* Kidney transplant recipients (KTRs) are at a higher risk of severe COVID-19 development. The course of the infection may vary. Long-term consequences for graft function are still being studied. We investigate whether the clinical course of SARS-CoV-2 infection among KTRs had a long-term effect on graft function. *Patients and method:* 128 KTRs with confirmed SARS-CoV-2 infection were included in the study. They were divided into two groups: mild (without the need for oxygen therapy; *n* = 91) and severe (with the need for oxygen therapy; *n* = 21). Baseline characteristics and medical data, especially creatinine level, estimated glomerular filtration rate (eGFR) CKD-EPI, and proteinuria, were analyzed. The main outcomes were the absolute and relative change in eGFR during the one-year follow-up after COVID-19. In the final models, sex, age, smoking, presence of diabetes mellitus (DM), and cardiovascular disease (CVD) were included. *Results:* KTRs with severe COVID-19 were older, more likely to smoke, and had DM and CVD more frequently. Our analysis reveals that COVID-19 severity was associated with a significantly more pronounced relative eGFR decline one year after recovery only in males [−13.94 (95% CI: −25.13 to −2.76, *p* = 0.015) percentage points]. One year after the disease onset, males with a severe course of the infection had a higher eGFR decline than those with a mild one. The COVID-19 severity did not affect eGFR loss in females. *Conclusions:* In KTRs suffering from COVID-19, deterioration of graft function was noticed. The eGFR decline was associated with disease severity and sex. It indicates a need for further research, observation, and preventive actions for KTRs, especially males.

## 1. Introduction

Kidney transplant recipients (KTRs) are a unique group of patients during the severe acute respiratory syndrome coronavirus 2 (SARS-CoV-2) pandemic. They are at a higher risk of severe coronavirus disease 2019 (COVID-19) development because of immunosuppression, pre-existing chronic kidney disease, and comorbidity [1]. Researchers from California found more COVID-19 incidents among KTRs in comparison to recipients of other organs [2]. A meta-analysis of studies in organ transplant patients conducted by Wen An et al. showed morbidity of 5.09%, intensive care unit (ICU) admissions at 21.23%, and mortality of 19.83% among KTRs [3,4]. On the other hand, the SARS-CoV-2 infection increases the risk of renal damage. The most common kidney injury associated with COVID-19 is acute tubular necrosis, which also affects vascular and glomerular systems. Pathophysiological mechanisms of COVID-19-related AKI may include hypovolemia, nephrotoxicity of drugs, an imbalanced renin–angiotensin–aldosterone system (RAAS) activation, an elevation of pro-inflammatory cytokines elicited by the viral infection and a direct viral injury. A procoagulant state causes renal thrombotic microangiopathy (TMA) or microvascular thrombosis, occluding vessels and contributing to the severity of AKI [3,5,6].

In the general population, COVID-19-associated acute kidney injury (AKI) was associated with a greater rate of eGFR decrease after discharge compared with AKI in patients without COVID-19 [7]. In parallel, KTRs with COVID-19 were at a higher risk of AKI, AKI requiring kidney replacement therapy, and graft loss [8]. Even 10.6% of patients hospitalized at ICU required dialysis or continuous renal replacement therapy, and 10.6% lost their grafts within 3 months of COVID-19 presentation [9]. There is also the risk of long-term complications in the form of post-COVID syndrome [10].

The severity of COVID-19 among KTRs ranged from a mild course to severe pneumonia, multi-organ failure, and death [11]. However, long-term consequences for graft function may differ depending on the clinical course of the disease. This knowledge may be of importance for transplant professionals and patients. Therefore, we undertook the study to investigate whether the clinical course of SARS-CoV-2 infection had a long-term effect on graft function.

## 2. Materials and Methods

### 2.1. Study Population

This longitudinal descriptive study was conducted in adult KTRs from the outpatient department in the university hospital in Gdańsk, Poland. They were retrospectively screened for COVID-19 diagnosis. A total of 128 patients with infection confirmed either with reverse-transcription polymerase chain reaction (RT-PCR), antigen (when the symptoms of illness occurred), or elevated SARS-CoV-2 IgG antibodies tests (checked after infection and before vaccination) between July 2020 and May 2021 were included in the study. They were not vaccinated. Following the aim of the study, patients were categorized into two groups during the analysis based on the course of the disease: mild (without the need for oxygen therapy) and severe (with the need for oxygen therapy). Non-survivors were assigned to a separate group and were not included in the main analyses. The criterion for the division was the need for oxygen therapy following the Polish recommendations for the management of SARS-CoV-2 infections of the Polish Society of Epidemiologists and Infectiologists from March 2020 [12]. We took February 2021 as the cut-off point when the new variant of a virus, called “British”, prevailed in our province, according to the website sarswpolsce.pl (accessed on 20 May 2022, created as a result of research by the Malopolska Center of Biotechnology of the Jagiellonian University as part of the SARS-CoV-2 virus genetic variability monitoring project) [13]. The etiology of chronic kidney disease in KTRs has been divided into 7 categories: glomerulonephritis (both primary and secondary), pyelonephritis, interstitial nephritis, diabetic nephropathy, hereditary nephropathies (e.g., polycystic kidney disease), congenital anomalies (e.g., vesicoureteral reflux), and of unknown origin, same as Duivenvoorden et al. [9].

### 2.2. Data Collection

Screening of people with COVID-19 proceeded as follows: information from the physician in the outpatient clinic or patient themself about COVID-19-related symptoms (such as fever, weakness, smell or taste disorder) was checked in the nationwide medical electronic data (gabinet.gov.pl; accessed on 20 April 2022) for laboratory confirmation. Baseline demographics, comorbidities, transplant details, and immunosuppressive therapies were extracted from the hospital’s electronic medical records system. Data on kidney function [serum creatinine level, estimated glomerular filtration rate (eGFR) calculated using the CKD-EPI 2009 equation, proteinuria (defined as any measurable amount of protein in the urine) at two time points (before and one year after COVID-19)] were retrospectively, manually collected by the study team using the hospital electronic medical records system. Survivors were asked about the course of the disease: symptoms at presentation, hospital admission, oxygen need, and implemented treatment using a unified questionnaire via telephone interview by trained staff. All data were collected from January 2021 to August 2022.

Ethics approval for the study was obtained at the Medical University of Gdańsk (NKBBN/2014/2021). The study is part of the ‘COVID-19 in Nephrology’ project focusing on the nephrological aspects of COVID-19 (COViNEPH).

### 2.3. Statistical Analysis

Descriptive statistics were used to report the characteristics of patients. Testing the normality of the distribution of collected data was performed using the Shapiro–Wilk test. Continuous variables with normal distribution were presented using mean and standard deviation (SD), whereas others used medians and interquartile ranges (IQR). Categorical variables were presented as a percentage share of the obtained data. Patient groups were compared using Student’s *t*-test, Mann–Whitney *U*, Pearson’s chi-squared (χ^2^), and Fisher’s exact tests.

The main outcomes were the absolute (the difference between the final and initial measurement) and relative (the difference between the final and initial measurement divided by the initial value and expressed as a percentage) change in eGFR of KTRs during the one-year follow-up after COVID-19. Linear regression models were used to test the difference between patients’ groups. To control for confounding, firstly, we adjusted models for sex (binary variable), age (continuous variable, centered), smoking (binary variable: never, current, or in the past), presence of diabetes mellitus (DM; binary variable), and presence of heart disease (CVD; binary variable). Due to the non-linear relation between the age and the eGFR change, we added an additional age-squared variable as a covariate. The summary results were estimated using the marginal effects approach (estimated marginal means). Since sex differences may be expected in the effect of the COVID-19 severity of eGFR loss [14], the final models also included an interaction term (sex × COVID-19 severity). We used the Akaike information criterion (AIC) to verify whether the inclusion of the interaction term increases the goodness of fit of the model while keeping its simplicity; a model with a lower AIC value was preferred [15]. The difference between AIC values (ΔAIC) was reported.

During the follow-up, three patients started dialysis. Therefore, data on their creatinine/eGFR levels were missing. To include two of them in the main analyses (there were *no data* on baseline creatinine in the case of the third one), we imputed data on serum creatinine levels equal to 5 mg/dL during dialysis, according to the literature [16].

The analyses were performed using R version 4.2.1 and its packages: gtsummary (1.6.2), dplyr (1.0.10), emmeans (1.8.2), ggplot2 (3.4.0), and ggplot_the_model script by Jeffrey A. Walker. [17].

## 3. Results

### 3.1. Patients

**Figure 1:**
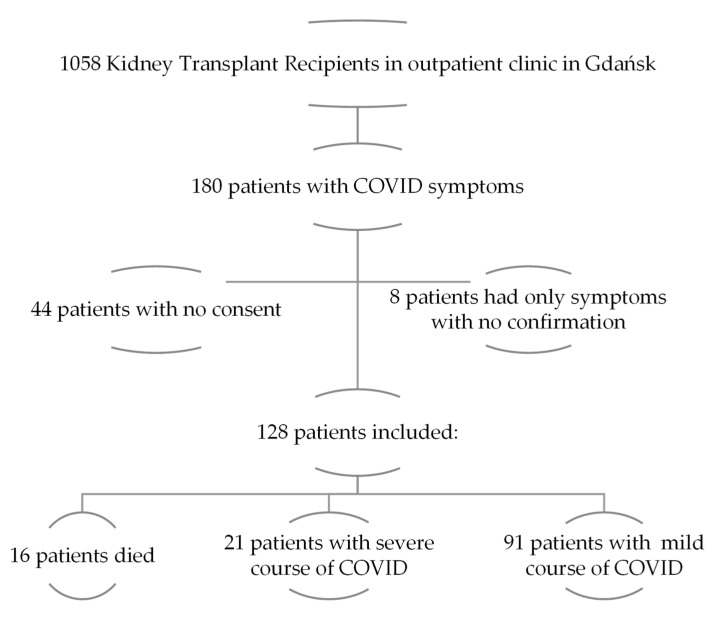
Flow diagram of patients recruited to the study.

Of 1058 KTRs screened, 180 patients had a history of COVID-19 symptoms, and 128 of them were included in the study. The enrollment process is shown in the flow diagram in Figure 1. Patients who died were excluded from statistical analyses. Therefore, the entire cohort was 112 patients. The median age was 51 (41–62) years, and 57.1% of the entire cohort were males. Hypertension was the most common comorbidity, present in 106 of 112 patients. In comparison with patients with a mild course of COVID-19, transplanted patients with severe COVID-19 were older [mean difference (MD): −8.19 (95% CI: −14.8 to 1.61) years, *p* = 0.015], were more likely to smoke currently or in the past, had more frequently DM, and were more frequently hospitalized due to SARS-CoV-2 infection. The median transplantation vintage was 6 (2–10) years, and a kidney came from a deceased donor in 93.7% of cases. The most common immunosuppression protocol included tacrolimus, mycophenolate, and steroids (54.5%). The detailed characteristics of all SARS-CoV-2 positive KTRs are shown in Table 1. The modifications of suppressive treatment in patients with COVID-19 can be found in Supplementary Material Table S4. We found that the proportion of never-smokers was lower among recruited males than females (51.6% vs. 89.6%, *p* < 0.001). There were no significant differences between sexes in the prevalence of hypertension (96.9% of males vs. 91.7% of females, *p* = 0.40), CVD (28.1% vs. 14.6%, *p* = 0.089), DM (26.6% vs. 18.8%, *p* = 0.33), or lung diseases (7.8% vs. 2.1%, *p* = 0.24).

### 3.2. Graft Function

**Figure 2:**
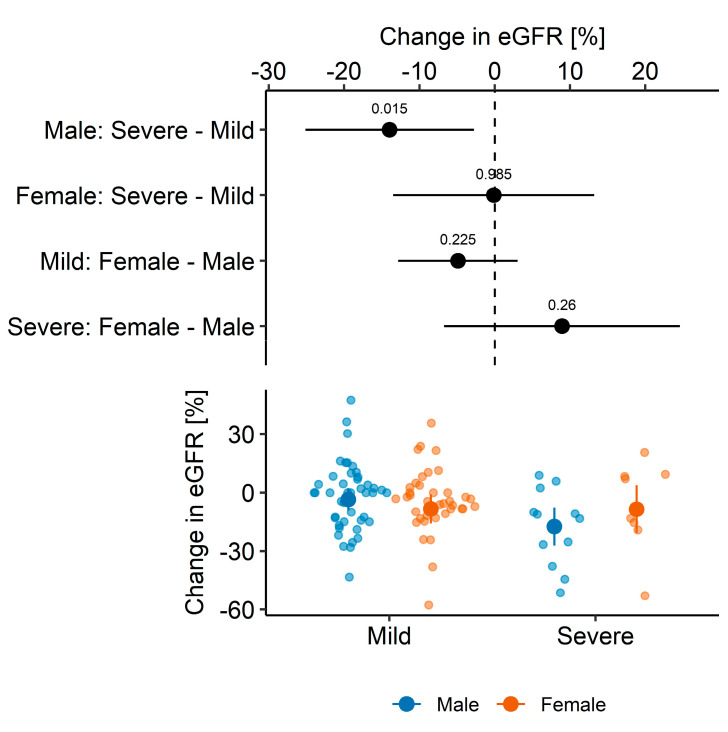
A relative change in eGFR—effect of COVID-19 severity adjusted for age, diabetes, heart disease, and smoking using estimated marginal means.

**Figure 3:**
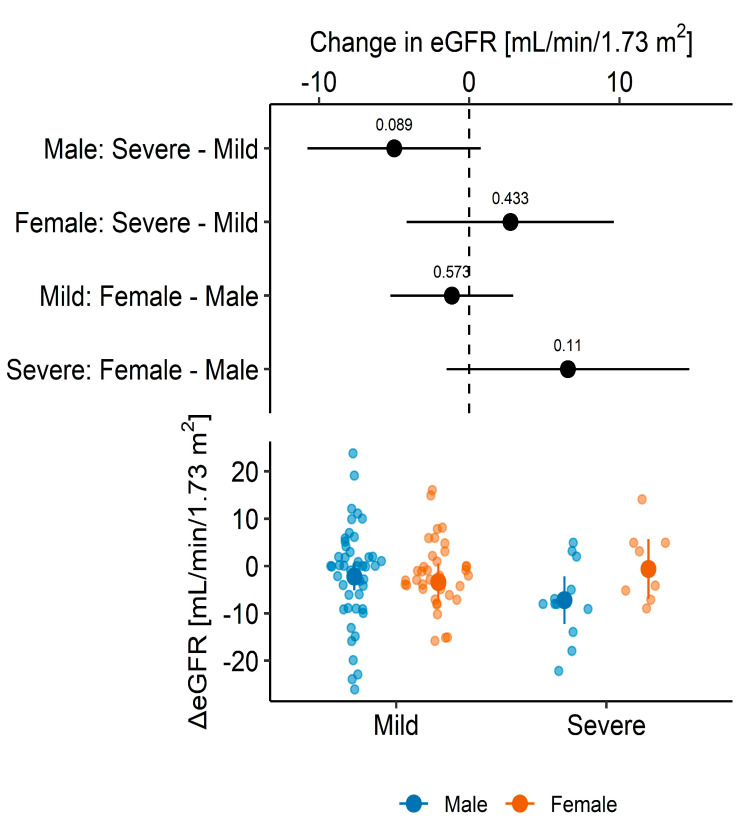
An absolute value of eGFR change—effect of COVID-19 severity adjusted for age, smoking, diabetes, and heart disease using estimated marginal means.

As expected, the function of the transplanted kidney was reduced during the one-year follow-up. Among 104 patients, the mean one-year change in eGFR was −2.32 (95% CI: −4.05 to −0.58, *p* = 0.009) mL/min/1.73 m^2^ that corresponds to a −5.02% (95% CI: −8.53% to −1.51%, *p* = 0.006) change in eGFR. When analyzed separately, in patients with severe course of COVID-19, the change was estimated to −4.35 (95% CI: −8.41 to −0.28, *p* = 0.037) mL/min/1.73 m^2^ that corresponds to a −13.45% (95% CI: −23.42% to −3.47%, *p* = 0.011) change in eGFR, whereas in patients with mild COVID-19, the change was not significant [mean absolute difference: −1.83 (95% CI: −3.78 to 0.11, *p* = 0.06) mL/min/1.73 m^2^; mean relative difference: −3.01% (95% CI: −6.64% to 0.61%, *p* = 0.10). Since these two groups of patients differ in factors that may confound the analysis, we tested whether COVID-19 severity is independently associated with more pronounced eGFR loss using multivariable models to adjust for identified and measured confoundings (see details in the Statistical analysis Section 2.3). Firstly, after adjustment (for sex, age, smoking, and presence of DM or CVD), no significant difference in the absolute/relative one-year change in eGFR was demonstrated in relation to the COVID-19 severity [contrast between estimated marginal means in patients with severe vs. mild infection: −1.86 (95% CI: −6.5 to 2.77, *p* = 0.43) mL/min/1.73 m^2^ that corresponds to −8.34% (95% CI: −17.3 to 0.62, *p* = 0.07)]. However, in the *post-hoc* analysis, we found that models that include interaction between COVID-19 severity and patient’s sex were more informative and more likely to represent reality than the models without this interaction (i.e., models with interaction term had lower AIC: ΔAIC_absolute ΔeGFR_ = 1.391; ΔAIC_relative ΔeGFR_ = 0.890). Compared to males with a mild course of COVID-19, males with a severe course of COVID-19 had significantly more pronounced relative eGFR decline during the follow-up: after controlling for confounders, the adjusted difference was equal to an average of −13.94 (95% CI: −25.13 to −2.76, *p* = 0.015) percentage points (Figure 2 and Figure 3). In contrast, no such difference in relative loss of eGFR was observed among females (*p* = 0.99). However, if the absolute change in eGFR over one year was analyzed (Figure 3), a significant difference was established in neither males [−5.00 (95% CI: −10.78 to 0.78, *p* = 0.09) mL/min/1.73 m^2^] nor females (*p* = 0.43). The estimated parameters of regression models used to calculate marginal means were shown in Supplementary Materials, Tables S1 and S2. 

Similar conclusions arise from the analysis of the change in creatinine level during follow-up: after controlling for confounders, higher COVID-19 severity was associated with a higher increase in serum creatinine level in males [0.41 (95% CI: 0.11 to 0.70) mg/dL, *p* = 0.007] but not in females [0.17 (95% CI: −0.18 to 0.52) mg/dL, *p* = 0.33], when compared to same-sex patients observed after a mild COVID-19 (Supplementary Materials, Figure S1 and Table S3).

Proteinuria was detected in 12 (10.3%) and 15 (14.7%) patients at the beginning and end of the study, respectively (missing data for 13 and 28 patients, respectively). During the follow-up, 8 patients developed proteinuria de novo; in the case of 6 patients, proteinuria persisted throughout the study, whereas remission of proteinuria was observed only in one patient (baseline proteinuria status unknown in the case of 1 patient with proteinuria at the follow-up). Unfortunately, such a small study size prohibited us from the analysis of factors affecting the risk of maintaining/emerging (de novo).

## 4. Discussion

Our study has revealed that COVID-19 severity may impact the loss of kidney graft function in a one-year follow-up in a sex-specific manner. We showed that one year after the disease onset, males with a severe course of the infection had a more pronounced relative eGFR decline than those with a mild one, whereas the severity of COVID-19 did not affect eGFR loss in females.

Our observation corresponds to the results of studies in the general population, where renal complications were significantly more likely in male patients [18]. Publications to date indicate possible explanations for the different courses and outcomes of COVID-19 depending on sex, which could be multifactorial: genetic, hormonal, immunological, racial, and psychosocial. For example, the spike protein of SARS-CoV-2 links with an ACE2 receptor, while the estradiol probably regulates ACE2 expression in airway epithelial cells, kidneys, and cardiac and adipose tissues [19].

Most of the studies on the function of transplanted kidneys published so far relate to the up-to-6-months follow-up period. In our previous preliminary report, we showed the lack of a significant change in the graft function 6 months after COVID-19 [20]. The percentage of survivors who received dialysis treatment during the COVID-19 follow-up in our study was similar to that of Demir et al. Turkish study (1.9% vs. 2.7%) [21], while in a Croatian study, only 2 of 267 patients progressed to kidney failure [22].

According to the Duivenvoorden et al. study, within 3 months of follow-up, graft failure was noted as rather rare among non-hospitalized and hospitalized KTRs not admitted to the ICU (<5%). However, even 10.6% of severely ill patients (admitted to the ICU) experienced loss of graft function. However, the sex of these patients was not specified [9]. In the study by Elec et al., no episode of acute rejection and graft loss were recorded after at least 2 months of follow-up [23]. In another study, a biopsy-proven acute rejection in COVID-19 patients was similar to the controls (1.1 and 1.0%, respectively) [24]. Indeed, when comparing kidney function in short-term observation post-discharge relative to that upon hospital admission, even a significant improvement was observed [25]. However, the long-term results are not known.

Data reported by Moein et al. revealed that KTRs exhibit no change in kidney function, as assessed by eGFR and serum creatinine level, after 12 months when compared with their pre-COVID-19 baseline values. However, in the study, neither sex nor the severity of the infection itself were taken into account. Additionally, the study also demonstrated that reducing immunosuppressive medication did not impact kidney allograft function when compared to the group in which immunosuppressive medication was continued at pre-COVID-19 levels [26]. However, it is important to note that a limitation of the study is the inclusion of vaccinated patients; the specific dosages of the medications being reduced and how this was performed were not specified.

In the study examining the consequences of COVID-19 at 2 years post-infection in the general population, significant differences were observed in both glomerular (eGFR, creatinine) and tubular (α1-microglobulin) kidney biomarkers between the non-severe and severe infection groups. The severe groups encompassed patients with severe illness (tachypnea, or decreased resting oxygen saturation, or decreased arterial partial pressure of oxygen/fraction of inspired oxygen ≤300 mmHg) and critical illness (patients with respiratory failure requiring mechanical ventilation, shock, or other organ failure necessitating intensive care unit). Advanced age and male sex were identified as independent risk factors for decreased eGFR after two years [27]. Both this study and our investigation indicate that the consequences of COVID-19 infection on kidney function may be long-lasting.

We can compare the current pandemic with the H1N1 influenza pandemic of 2009. The study [28] presented that the incidence of acute renal failure was higher among KTRs in comparison to non-immunocompromised patients (40.9% vs. 17%). However, there is a scarcity of follow-up studies on kidney function.

Our study has several limitations. Firstly, COVID-19 could have been underreported due to the lack of testing for all outpatients for SARS-CoV-2 infection and asymptomatic cases. The small patient population is likely to result in statistically significant findings in the form of only a relative reduction in eGFR. Consequently, we did not have a control group because other patients could have been infected with COVID-19 in the following months. Furthermore, in May 2022, mass control of infection in Poland was discontinued, and the necessity of quarantine and isolation was lifted. Another limitation was the limited access to comprehensive medical documentation of the patients. The absence of detailed data regarding treatment, especially for hospitalized patients, modifications to immunosuppressive therapy, antibiotic doses, duration and doses of administered oxygen therapy, as well as laboratory test results, restricted the possibility of statistically analyzing the impact of treatment on the function of the transplanted kidney. Additionally, patients in various Pomeranian hospitals were in specially designated COVID units where both conservative and intensive care treatments were administered. Therefore, the results need to be validated by larger prospective cohort studies.

## 5. Conclusions

In conclusion, graft function was decreased over time in all KTRs. COVID-19 severity was associated with a higher increase in creatinine levels and greater relative eGFR decline in males. In the case of patients with a mild course of the disease, sex did not matter.

Our finding indicates a need for further research and observation of KTRs. Males should be under special care and preventive actions; for example, vaccinations should go to male transplanted patients.

## Figures and Tables

**Table 1:** Characteristics of the study population.

	ALL	MILD	SEVERE	*p* Value ^1^
*n*	112	91	21	
Age years, median (IQR)	51 (41–62)	50 (38–61)	58 (49–67)	0.022
Male sex, *n* (%)	64 (57.1)	52 (57.1)	12 (57.1)	>0.99
BMI, median (IQR) *No data, n*	25 (22–29) 1	24 (22–29) 1	26 (23–32) 0	0.19
Comorbidities *n* (%)				
Hypertension	106 (94.6)	85 (93.4)	21 (100)	0.59
Diabetes mellitus	26 (23.2)	16 (17.6)	10 (47.6)	0.008
Heart disease	25 (22.3)	17 (18.7)	8 (38.1)	0.079
Lung disease	6 (5.4)	6 (6.6)	0 (0.0)	0.59
Smoking, *n* (%)				0.028
Current or in the past	36 (32.1)	25 (27.5)	11 (52.4)	
Never	76 (67.9)	66 (72.5)	10 (47.6)	
Primary nephropathy *n* (%)				0.80
Unknown or other	36 (32.4)	29 (32.2)	7 (33.3)	
Glomerulonephritis	36 (32.4)	31 (34.4)	5 (23.8)	
Pyelonephritis	2 (1.8)	2 (2.2)	0 (0)	
Interstitial nephritis	4 (3.6)	3 (3.3)	1 (4.8)	
Congenital anomalies	5 (4.5)	4 (4.4)	1 (4.8)	
Diabetic nephropathy	8 (7.2)	7 (7.8)	1 (4.8)	
Hereditary nephropathies	20 (18.0)	14 (15.6)	6 (28.6)	
*No data*, *n*	1	1	0	
Transplantation vintage years, median (IQR)	6 (2–10)	6 (2–10)	6 (3–11)	0.55
Deceased donor, *n* (%)	105 (93.7)	84 (92.3)	21 (100)	0.34
Medications, *n* (%)				
ACEi	31 (27.9)	23 (25.6)	8 (38.1)	0.25
ARB	10 (9.0)	7 (7.8)	3 (14.3)	0.40
Vitamin D	60 (54.1)	50 (55.6)	10 (47.6)	0.51
*No data*, *n*	1	1	0	
Immunosuppressive agents, *n* (%)				>0.99
TAC+MMF/MPS + steroids	61 (54.5)	49 (53.8)	12 (57.1)	
CyA+MMF/MPS + steroids	26 (23.2)	21 (23.1)	5 (23.8)	
CNI+steroids, without antiproliferative	14 (12.5)	12 (13.2)	2 (9.5)	
Other protocols	11 (9.8)	9 (9.9)	2 (9.5)	
Hospitalized, *n* (%) *No data*, *n*	46 (41.1) 0	26 (28.6) 0	20 (95.2) 0	<0.001
Duration of hospitalization in days, median (IQR) Valid data/*No data, n*	12 (9–19) 39/73	10 (8–14) 22/69	14 (12–26) 17/4	0.027
SARS-CoV-2 variants, *n* (%)				>0.99
Wild-type	80 (71.4)	65 (71.43)	15 (71.4)	
British variant	32 (28.6)	26 (28.6)	6 (28.6)	

^1^ Pearson’s chi-squared test; Wilcoxon rank sum test; Fisher’s exact test. BMI—body mass index; ACEi—angiotensin-converting-enzyme inhibitors; ARB—angiotensin receptor blockers; TAC—Tacrolimus; MMF—mycophenolate mofetil; MPS—mycophenolate sodium; CyA—cyclosporin A; CNI—calcineurin inhibitor; SARS-CoV-2—severe acute respiratory syndrome coronavirus 2.

## Data Availability

Detailed data are available on request from the corresponding author.

## Supplementary Materials

The following supporting information can be downloaded at: https://www.mdpi.com/1648-9144/60/1/26/s1, Figure S1: An absolute change in serum creatinine—effect of COVID-19 severity adjusted for age, smoking, diabetes, heart disease. Table S1: Linear regression model of the absolute eGFR change. Table S2: Linear regression model of the relative eGFR change. Table S3: Linear regression model of the creatine change. Table S4: Modification of immunosuppression in patients with COVID-19 according to the course of the infection.
