# Supplementary material for: Effect of COVID-19 on Kidney Graft Function One Year after Onset
Source: Medicina (Kaunas). 2023 Dec 23;60(1):26. doi: 10.3390/medicina60010026 (PMC11154522; doi:10.3390/medicina60010026)
Supplement: Supplementary file 1 [file medicina-60-00026-s001.zip › medicina-2756075-supplementary.pdf]

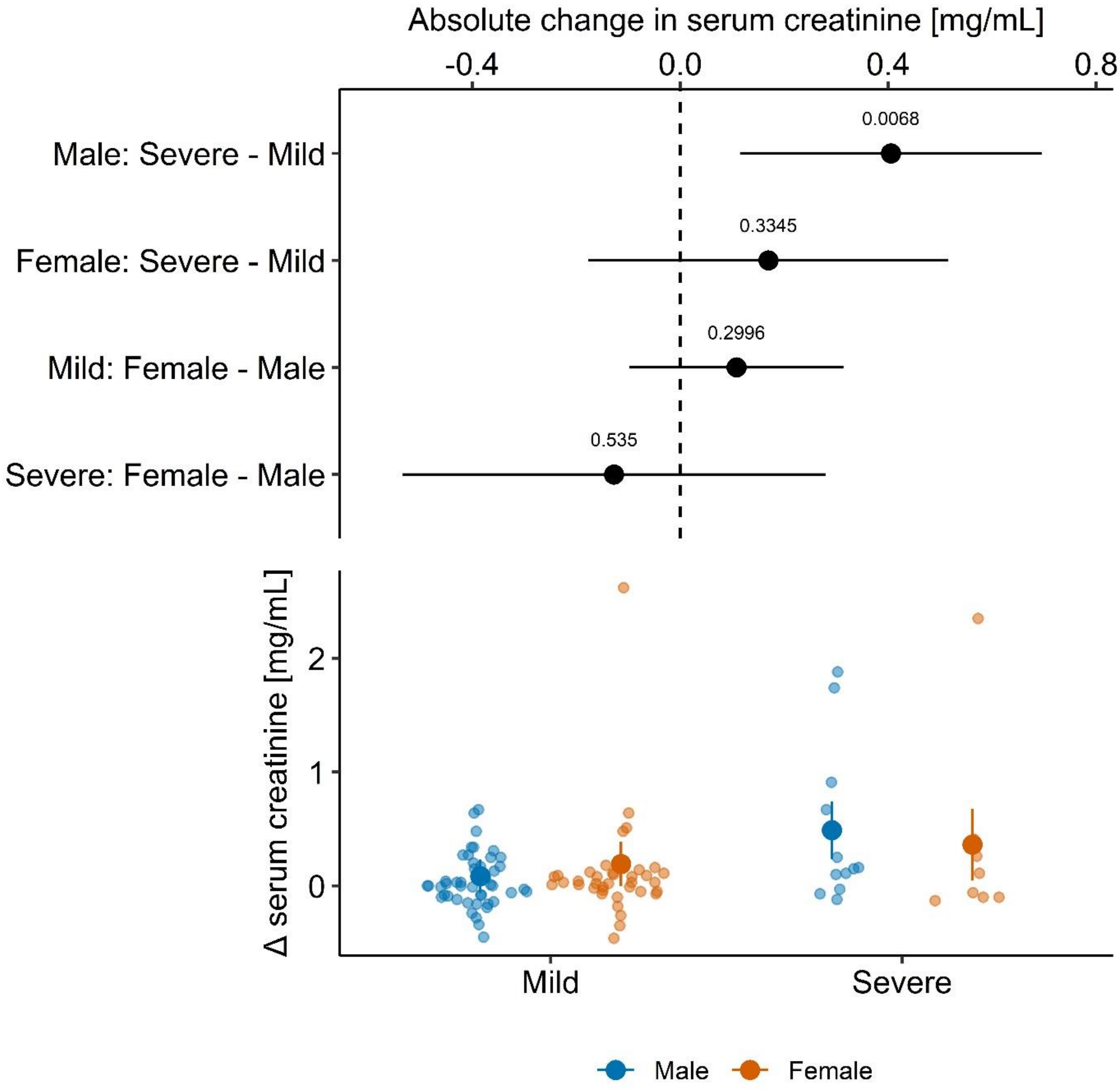

Figure S1. An absolute change in serum creatinine - effect of COVID-19 severity adjusted for age, smoking, diabetes, heart disease

Table S1. Linear regression model of the absolute eGFR change.

**Predictor**	**Coefficient estimate**	**Std. error**	***P* value**
Severe COVID-19 course [a]	−5.000	2.909	0.089
Female sex [b]	−1.165	2.061	0.573
Smoking [c]	−1.005	0.080	0.630
Age (centered)	−0.043	0.075	0.568
$[\text{Age (centered)}]^2$	−0.011	0.004	0.004
Diabetes mellitus	0.190	2.244	0.933
Heart disease	−2.482	2.276	0.278

Interaction<br>sex × COVID severity	7.735	4.359	0.079
Intercept	1.524	1.917	0.43

[a] severe *versus* mild (where 'severe' indicates the necessity for any form of oxygen therapy); [b] female *versus* male; [c] either current or in the past *versus* never

Table S2. Linear regression model of the relative eGFR change.

**Predictor**	**Coefficient estimate**	**Std. error**	***P* value**
Severe COVID-19 course [a]	−13.944	5.636	0.015
Female sex [b]	−4.877	3.993	0.225
Smoking [c]	−1.489	4.031	0.713
Age (centered)	−0.152	0.145	0.295
[Age (centered)]^2	−0.026	0.007	0.001
Diabetes mellitus	-0.086	4.347	0.984
Heart disease	−7.119	4.408	0.110
Interaction<br>sex × COVID severity	13.818	8.446	0.105
Intercept	5.680	3.714	0.130

[a] severe *versus* mild (where 'severe' indicates the necessity for any form of oxygen therapy); [b] female *versus* male; [c] either current or in the past *versus* never

Table S3. Linear regression model of the creatine change.

**Predictor**	**Coefficient estimate**	**Std. error**	***P* value**
Severe COVID-19 course [a]	0.405	0.146	0.007
Female sex [b]	0.108	0.104	0.30
Smoking [c]	−0.035	0.105	0.741
Age (centered)	0.004	0.004	0.289
[Age (centered)]^2	0.001	0.0002	0.003
Diabetes mellitus	-0.017	0.113	0.882
Heart disease	0.212	0.114	0.067
Interaction<br>sex × COVID severity	-0.236	0.219	0.285
Intercept	−0.103	0.096	0.289

[a] severe *versus* mild (where 'severe' indicates the necessity for any form of oxygen therapy); [b] female *versus* male; [c] either current or in the past *versus* never

Table S4. Modification of immunosuppression in patients with COVID-19 according to the course of the infection

	ALL	MILD	SEVERE	*P* value
N	112	91	21	

Management of antiproliferative treatment [a], n (%)				
No change	42 (48.3)	39 (52.0)	3 (25.0)	0.082
Reduced dosage / discontinuation	45 (51.7)	36 (48.0)	9 (75.0)	
Not applicable/No data	25	16	9	
Management of steroids treatment, n (%)				
No change	75 (75.8)	70 (84.3)	5 (31.3)	<0.001
Reduced dosage	7 (7.1)	4 (4.8)	3 (18.8)	
Increased dosage	17 (17.2)	9 (10.8)	8 (50.0)	
Not applicable/No data	13	8	5	

[a] Antiproliferative drugs: MMF, MPS, AZA, mTOR inhibitors (everolimus, sirolimus)
